# Patterns of Co-Occurrence of Chronic Disease Among Older Adults in Tokyo, Japan

**DOI:** 10.5888/pcd16.180170

**Published:** 2019-01-31

**Authors:** Seigo Mitsutake, Tatsuro Ishizaki, Chie Teramoto, Sayuri Shimizu, Hideki Ito

**Affiliations:** 1 Human Care Research Team, Tokyo Metropolitan Institute of Gerontology, Tokyo, Japan; 2 Division of Health Sciences and Nursing, Graduate School of Medicine, The University of Tokyo, Tokyo, Japan; 3 Institute of Health Economics and Policy, Tokyo, Japan; 4 Tokyo Metropolitan Geriatric Hospital and Institute of Gerontology, Tokyo, Japan

## Abstract

**Introduction:**

Multimorbidity, the co-occurrence of 2 or more disorders in a patient, can complicate treatment planning and affect health outcomes. Improvements in prevention and management strategies for patients with 3 or more or more co-occurring chronic diseases requires an understanding of the epidemiology of common 3-way disease patterns and their interactions. Our study aimed to describe these common 3-way disease patterns and examine the factors associated with the co-occurrence of 3 or more diseases in elderly Japanese patients.

**Methods:**

We included all Japanese citizens aged 75 or older living in Tokyo who used medical care between September 2013 and August 2014 (N = 1,311,116) in our analysis. The 15 most common 3-way patterns of 22 target diseases according to sex and age were identified from among all possible combinations by using an anonymized medical claims database. We examined the associations of sociodemographic characteristics and health care use with the presence of 1 or 2 co-occurring diseases and 3 or more co-occurring diseases by using multinomial logistic regression.

**Results:**

Approximately 65% of patients had 3 or more co-occurring diseases. The most common 3-way pattern was hypertension, coronary heart disease, and peptic ulcer disease in men (12.4%) and hypertension, dyslipidemia, and peptic ulcer disease in women (12.8%). The prevalence of 3 or more diseases was positively associated with men, patients aged 85 to 90, the use of home medical care services, the number of outpatient facilities visited, and hospital admissions.

**Conclusion:**

The common 3-way disease patterns and multimorbidity factors identified in our study may facilitate the recognition of high-risk patients and support the development of clinical guidelines for multimorbidity.

**MEDSCAPE CME**

Medscape, LLC is pleased to provide online continuing medical education (CME) for this journal article, allowing clinicians the opportunity to earn CME credit.

In support of improving patient care, this activity has been planned and implemented by Medscape, LLC and *Preventing Chronic Disease*. Medscape, LLC is jointly accredited by the Accreditation Council for Continuing Medical Education (ACCME), the Accreditation Council for Pharmacy Education (ACPE), and the American Nurses Credentialing Center (ANCC), to provide continuing education for the healthcare team.

Medscape, LLC designates this Journal-based CME activity for a maximum of 1.00 *AMA PRA Category 1 Credit(s)™*. Physicians should claim only the credit commensurate with the extent of their participation in the activity.

All other clinicians completing this activity will be issued a certificate of participation. To participate in this journal CME activity: (1) review the learning objectives and author disclosures; (2) study the education content; (3) take the post-test with a 75% minimum passing score and complete the evaluation at http://www.medscape.org/journal/pcd; (4) view/print certificate.

**Release date: January 31, 2019; Expiration date: January 31, 2020**

**Learning Objectives**

Upon completion of this activity, participants will be able to:

1. Distinguish the average number of chronic illnesses per patient in the current study
2. Assess the most common 3 diseases found together among women in the current study
3. Assess the most common 3 diseases found together among men in the current study
4. Evaluate risk factors for multiple chronic illnesses in the current study

**EDITOR**

Rosemarie Perrin

Editor, *Preventing Chronic Disease*

Disclosure Rosemarie Perrin has disclosed no relevant financial relationships.

**CME AUTHOR**

Charles P. Vega, MD

Clinical Professor, Health Sciences

Department of Family Medicine

University of California, Irvine School of Medicine

Disclosure: Charles P. Vega, MD, has disclosed the following relevant financial relationships:

Served as an advisor or consultant for: Johnson & Johnson Pharmaceutical Research & Development, LLC; Shire Pharmaceuticals; Sunovion Pharmaceuticals Inc.

Served as a speaker or a member of a speakers bureau for: Shire Pharmaceuticals

**AUTHORS**

Seigo Mitsutake, PT, PhD

Human Care Research Team, Tokyo Metropolitan Institute of Gerontology, Tokyo, Japan

Disclosure: Seigo Mitsutake, PT, PhD, has disclosed no relevant financial relationships.

Tatsuro Ishizaki, MD, PhD, MPH

Human Care Research Team, Tokyo Metropolitan Institute of Gerontology, Tokyo, Japan

Disclosure: Tatsuro Ishizaki, MD, PhD, MPH, has disclosed no relevant financial relationships.

Chie Teramoto, RN, PHN, PhD

Human Care Research Team, Tokyo Metropolitan Institute of Gerontology, Tokyo, Japan. Division of Health Sciences and Nursing, Graduate School of Medicine, The University of Tokyo, Tokyo, Japan

Disclosure: Chie Teramoto, RN, PHN, PhD, has disclosed no relevant financial relationships.

Sayuri Shimizu, PhD

Institute of Health Economics and Policy, Tokyo, Japan

Disclosure: Sayuri Shimizu, PhD, has disclosed no relevant financial relationships.

Hideki Ito, MD, PhD

Tokyo Metropolitan Geriatric Hospital and Institute of Gerontology, Tokyo, Japan.

Disclosure: Hideki Ito, MD, PhD, has disclosed no relevant financial relationships.

## Introduction

Multimorbidity, the co-occurrence of 2 or more disorders in a patient, can complicate treatment planning and affect health outcomes. Effective strategies are therefore needed to manage these conditions (1,2). Multimorbidity is most common in older adults (3–5) and can reduce functional status, quality of life, and survival (5,6). Current clinical guidelines generally focus on single diseases, rarely address the co-occurrence of 2 diseases, and almost never provide protocols for the co-occurrence of 3 or more diseases (7). Management strategies for multimorbidity often involve complex polypharmacy, which can increase treatment burden and the risk of adverse drug events.

Understanding the epidemiology of multimorbidity is critical to improving its prevention and management (2,7–10). The US Department of Health and Human Services has emphasized the importance of identifying common patterns in the occurrence of 2 or 3 diseases to guide the development of specific interventions for drug interactions (2). Approximately half of all adults aged 75 or older have 3 or more co-occurring chronic diseases (3), and prior knowledge of common 3-way disease patterns may enable clinicians to anticipate and manage multiple drug interactions. However, previous studies have generally focused on common 2-way disease patterns, and few studies have investigated 3-way patterns (7,8,11–13).

The identification of common disease patterns among older adults with 3 or more co-occurring chronic diseases can support the development of useful clinical guidelines for multimorbidity. Moreover, the elucidation of factors associated with the co-occurrence of 3 or more diseases may enable policy makers to identify and improve aspects of the health care system designed to treat multimorbidity. However, few studies have investigated these factors in Japan. Our study aimed to describe the common 3-way chronic disease patterns and to examine the factors associated with the co-occurrence of 3 or more chronic diseases among Japanese citizens aged 75 or older.

## Methods

### Database and study sample

We extracted data for this observational study from a large-scale, anonymized medical claims database obtained from the Tokyo Extended Association of Medical Care System for the Latter-Stage Elderly People, which manages the medical insurance program for Tokyo residents aged 75 or older (14). Enrollment in this insurance program is mandatory for each Japanese citizen on her or his 75th birthday (14). This database therefore encompasses data on all citizens aged 75 or older living in Tokyo, the capital city of Japan.

Data included patient-level sociodemographic characteristics, treatments, medical facilities used, drugs prescribed, and diagnoses made during clinical encounters for insurance claims. We recorded diagnoses as *International Statistical Classification of Diseases and Related Health Problems*, 10th Revision (ICD-10) codes. We obtained data on adults aged 75 or older who had received health care at a hospital or other medical institution between September 1, 2013, and August 31, 2014 (N = 1,311,116). Total outpatient medical expenditure information was also collected. Costs were converted from Japanese yen to US dollars by using the exchange rate of August 2014 ($1 = 103 yen) (15).

### Definitions of chronic diseases and other variables

On the basis of prior studies (3,8,16), we selected 19 chronic diseases that are common in older (defined as ≥75 y) Japanese adults that can be identified by specific drug classes. In addition, we selected 3 chronic diseases (cancer, cerebrovascular accident, and coronary heart disease) that are common causes of death among older adults in Japan. Because the diagnoses recorded in claims data were not verified by clinically trained professionals (13,17), the use of these diagnoses alone may not be sufficiently robust. To minimize the influence of erroneous diagnoses, we identified 19 of the diseases by using a combination of ICD-10 codes and the recorded administration of drug classes specifically prescribed to treat these diseases in Japan. We identified patients as having 1 of these 19 diseases if their claims data from outpatient care showed the recorded administration of a relevant drug class in the same or following month as the target diagnosis. However, the drug classes commonly prescribed to treat cancer, cerebrovascular accident, and coronary heart disease are not sufficiently specific to support their identification; these diseases were identified by using only ICD-10 codes.

Sociodemographic variables included sex and age groups (75–79 y, 80–84 y, 85–89 y, 90–94 y, 95–99 y, and ≥100 y). Household income was divided into 2 categories according to resident taxable income: high household income (resident taxable household income ≥$14,078) and nonhigh household income (resident taxable household income <$14,078) (14). Health care variables included the use or nonuse of home medical care services, number of outpatient facilities visited, and number of hospital admissions during the study period. We identified the use of home medical care services through relevant records in the claims data. We divided the number of outpatient facilities visited into 5 categories (1, 2, 3, 4, and ≥5 facilities), and divided the number of admissions into 4 categories (0, 1, 2, and ≥3 admissions).

### Analysis

We used χ^2^ tests to compare the prevalences of the 22 diseases among sex and age groups. We first generated all possible 3-way combinations of the 22 diseases and identified the 15 most common patterns according to sex and age group (8). People with 4 or more co-occurring chronic diseases provided more than one 3-way pattern. For example, a person with hypertension, dyslipidemia, diabetes, and osteoporosis would have the following 3-way combinations: pattern 1, hypertension-dyslipidemia-diabetes; pattern 2, hypertension-dyslipidemia-osteoporosis; pattern 3, hypertension-diabetes-osteoporosis; and pattern 4, dyslipidemia-diabetes-osteoporosis.

We performed a multinomial logistic regression analysis to examine the associations of sociodemographic and health care variables with the presence of 1 or 2 co-occurring diseases and 3 or more co-occurring diseases after adjusting for all other covariates. The dependent variable comprised the following categories: no disease, the presence of 1 or 2 co-occurring diseases, and the presence of 3 or more co-occurring diseases. The independent variables included sociodemographic and health care variables. We calculated adjusted odds ratios and 95% confidence intervals for each variable. In all analyses, *P* values were 2-sided; those under .05 were considered significant. We conducted all analyses using SPSS version 23.0 (IBM Corp).

We obtained approval of the study protocol from the Ethics Committee of the Tokyo Metropolitan Geriatric Hospital and Institute of Gerontology (Approval no: 26_1437). We performed all procedures in accordance with the Ethical Guidelines for Medical and Health Research Involving Human Subjects established by the Japanese government.

## Results

A total of 1,311,116 patients were included in the analysis (Table 1). The mean age was 81.3 (standard deviation [SD], 5.4 y), and 806,059 (61.5%) were women. A total of 847,304 (65%) had 3 or more co-occurring chronic diseases and 1,051,621 (80.2%) had 2 or more co-occurring chronic diseases.

**Table 1:** Characteristics of Participants (N = 1,311,116), Study of Patterns of Co-Occurrence of Chronic Diseases Among People Aged ≥75 in Tokyo, Japan, September 2013–August 2014

Characteristic	N (%)^a^
**Sex**	
Men	505,057 (38.5)
Women	806,059 (61.5)
**Age, y**	
75–79	597,696 (45.6)
80–84	378,177 (28.8)
85–89	218,274 (16.6)
90–94	88,704 (6.8)
95–99	24,172 (1.8)
≥100	4,093 (0.3)
Income^b^	
Nonhigh income	1,122,641 (85.6)
High income	188,475 (14.4)
**Use of home medical care services**	
No	1,211,104 (92.4)
Yes	100,012 (7.6)
**Number of outpatient facilities visited**	
1	265,955 (20.3)
2	317,017 (24.2)
3	273,019 (20.8)
4	193,577 (14.8)
≥5	261,548 (19.9)
**Number of hospital admissions**	
0	1,012,827 (77.2)
1	186,212 (14.2)
2	66,339 (5.1)
≥3	45,738 (3.5)
**Number of chronic diseases**	
0	99,981 (7.6)
1	159,514 (12.2)
2	204,317 (15.6)
3	224,148 (17.1)
4	209,506 (16.0)
5	167,817 (12.8)
6	116,581 (8.9)
7	68,967 (5.3)
≥8	60,285 (4.6)

a Percentages may not total 100 because of rounding.

b Nonhigh income = resident taxable household income <$14,078); high income = resident taxable household income ≥$14,078.

**Figure 1:**
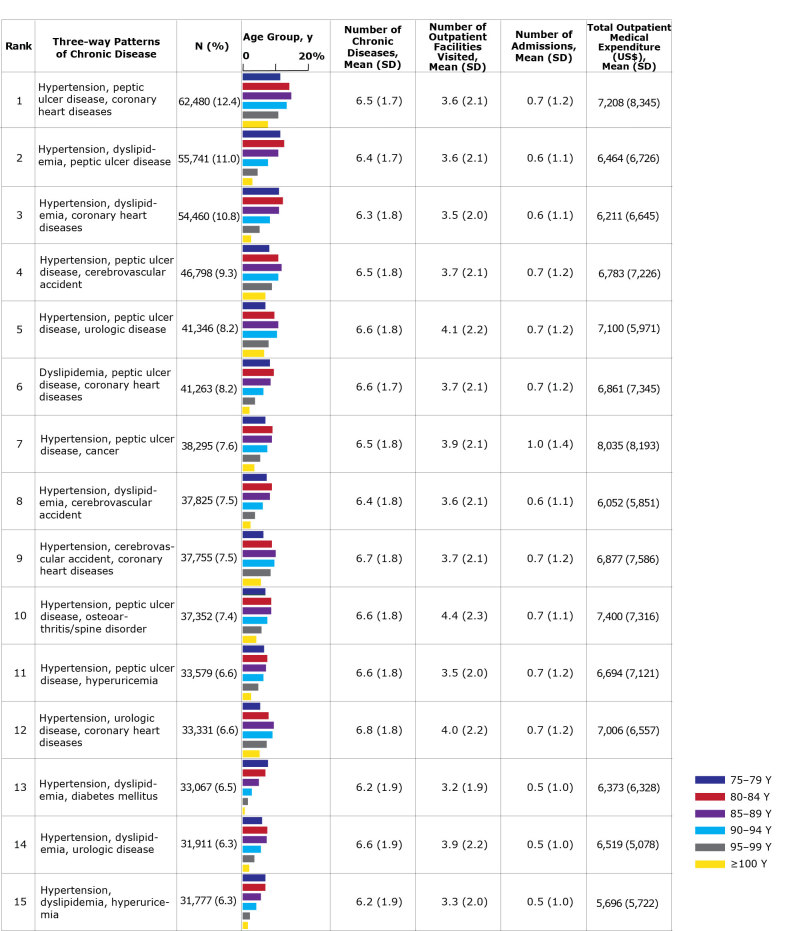
The 15 most common 3-way chronic disease patterns among men aged 75 or older in Tokyo, Japan (N = 505,057) by rank and age group with average number of chronic diseases and annual number of outpatient facilities visited, hospital admissions, and outpatient medical expenditures. Percentages do not total 100 because of rounding. Source: Tokyo Extended Association of Medical Care System for the Latter-Stage Elderly People (14). Abbreviation: SD, standard deviation.

**Figure 2:**
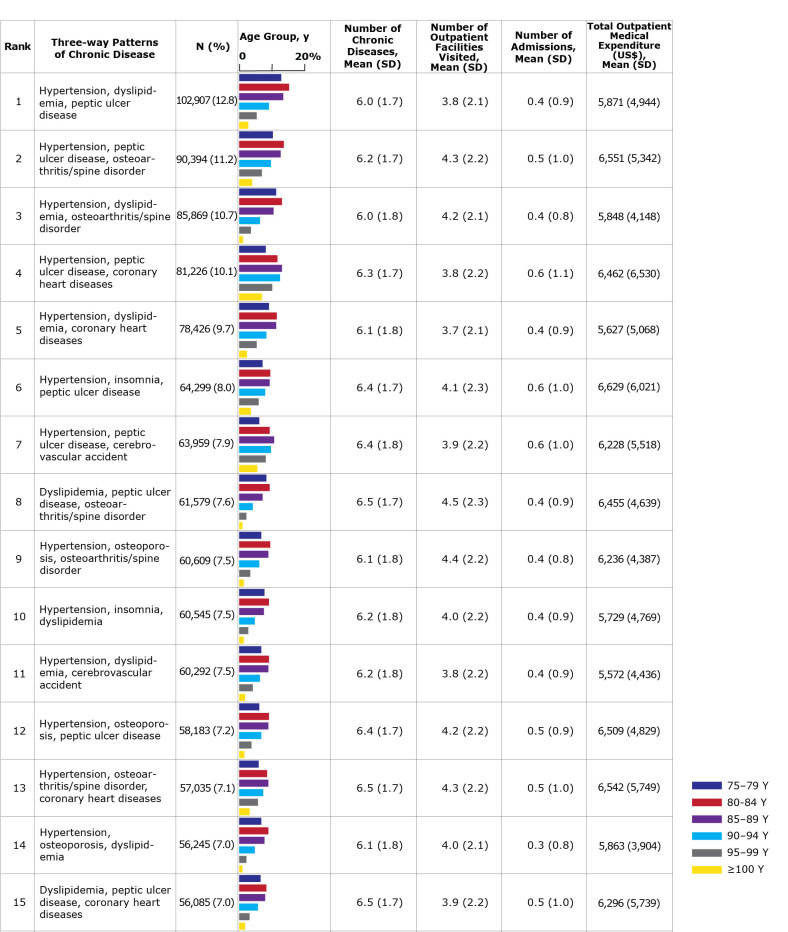
The 15 most common 3-way chronic disease patterns among women aged 75 or older in Tokyo, Japan (n = 806,059) by rank and age group with average number of chronic diseases and annual number of outpatient facilities visited, hospital admissions, and outpatient medical expenditures. Percentages do not total 100 because of rounding. Source: Tokyo Extended Association of Medical Care System for the Latter-Stage Elderly People (14). Abbreviation: SD, standard deviation.

The χ^2^ tests showed that the prevalences of 21 chronic diseases differed significantly between men and women, with anemia being the exception (Table 2). The prevalences of all diseases were significantly different among the age groups for each sex (Figures 1 and 2). The most common pattern among men was hypertension, coronary heart disease, and peptic ulcer disease; among women, the most common pattern was hypertension, dyslipidemia, and peptic ulcer disease. In both sexes, patterns frequently included hypertension, dyslipidemia, or peptic ulcer disease. However, large variations were observed between men and women in the prevalence of patterns that included urologic disease, osteoporosis, or osteoarthritis/spine disorder. Urologic disease was present in 3 of the patterns in men, but absent from the patterns in women. In contrast, osteoporosis and osteoarthritis/spine disorder appeared more frequently in women than in men. The prevalence of many 3-way disease patterns was lower in people aged 90 or older than those who were younger. Many people with common 3-way disease patterns also had other diseases. The mean number of chronic diseases per person ranged from 6.2 to 6.8 in men and 6.0 to 6.5 in women. The mean number of outpatient facilities visited was highest in men with co-occurring hypertension, peptic ulcer disease, and osteoarthritis/spine disorder, and highest in women with co-occurring dyslipidemia, peptic ulcer disease, and osteoarthritis/spine disorder. The mean total outpatient annual medical expenditure per patient was $4,389 for men and $4,089 for women. These expenditures also varied depending on 3-way disease pattern ($5,696–$8,035 in men and $5,572–$6,629 in women).

**Table 2:** Prevalence of 22 Common Chronic Diseases by Age Group Among Participants (N = 1,311,116), Study of Patterns of Co-Occurrence of Chronic Diseases Among People Aged ≥75 in Tokyo, Japan^a^

Chronic Disease	Men, y (n = 505,057)							Women, y (n = 806,059)						
	75–79	80–84	85–89	90–94	95–99	≥100	All	75–79	80–84	85–89	90–94	95–99	≥100	All
Hypertension	58.6	61.2	60.0	57.1	51.4	40.7	59.4	54.2	62.0	63.9	60.6	53.8	43.6	58.6
Peptic ulcer disease	35.0	39.2	39.4	38.2	32.7	29.8	37.0	34.7	39.6	39.9	37.8	34.2	28.6	37.3
Dyslipidemia	31.5	30.9	26.4	20.1	12.7	9.1	29.9	43.1	41.9	34.7	24.5	15.6	8.6	38.9
Coronary heart disease	25.5	31.0	33.4	33.6	33.1	33.5	28.7	19.5	25.5	29.4	31.0	31.4	30.0	24.3
Urologic disease	22.1	28.6	32.2	32.6	29.2	23.9	26.0	6.3	8.3	8.8	8.2	7.0	4.7	7.5
Cerebrovascular accident	21.3	27.7	31.9	32.9	32.3	29.6	25.4	16.6	23.2	27.3	28.5	28.7	26.7	21.7
Cancer	23.0	27.2	27.1	23.8	20.5	16.0	24.8	12.0	12.5	12.0	9.9	8.2	7.3	11.9
Osteoarthritis/spine disorder	20.2	23.1	23.1	21.4	19.4	15.8	21.5	32.5	35.5	31.6	25.1	20.5	13.4	32.2
Diabetes	20.2	18.6	14.4	10.4	8.3	4.2	18.3	12.4	12.2	11.0	8.4	5.8	3.2	11.5
Hyperuricemia	18.2	18.2	17.2	16.3	13.5	11.3	17.9	3.6	5.0	6.1	6.3	5.5	4.6	4.7
Insomnia	16.0	19.2	20.6	19.4	17.3	12.6	17.8	22.7	25.1	23.5	21.0	18.1	14.1	23.2
Chronic obstructive pulmonary disease	9.4	10.9	11.4	10.5	9.7	10.4	10.2	8.2	8.2	7.7	7.6	8.1	9.2	8.1
Cataract/glaucoma	9.3	10.9	11.5	10.5	8.7	7.6	10.1	11.3	12.8	12.1	9.9	7.3	5.3	11.6
Atrial fibrillation	8.9	10.5	11.5	10.8	8.2	5.7	9.8	4.1	5.8	7.1	6.8	5.5	3.4	5.4
Dementia	3.8	8.4	13.4	14.5	12.3	9.3	7.1	4.7	11.2	16.9	17.8	13.1	7.4	10.0
Anemia	3.6	5.2	6.8	7.8	8.5	7.7	4.7	3.0	4.9	6.8	7.9	7.7	7.4	4.8
Osteoporosis	2.6	4.4	5.8	5.8	5.8	4.2	3.8	21.1	25.3	23.6	18.2	11.9	6.2	22.2
Depression	2.2	2.7	2.8	2.5	1.8	1.2	2.5	4.4	5.3	5.1	4.2	3.2	1.9	4.7
Parkinson’s disease	1.8	2.5	2.5	2.0	1.5	0.8	2.1	1.7	2.3	2.4	2.0	1.1	0.9	2.0
Epilepsy	2.0	2.3	2.1	1.9	1.5	1.2	2.1	1.7	2.0	2.0	1.9	1.6	0.8	1.9
Hypthyroidism	1.1	1.6	1.9	2.3	2.9	2.9	1.5	2.6	2.9	3.0	2.9	2.4	2.0	2.8
Rheumatoid arthritis	0.7	0.6	0.5	0.4	0.2	0.0	0.6	1.9	1.6	1.2	0.7	0.3	0.1	1.5

a χ^2^ tests showed that the prevalences of 21 chronic diseases were significantly different between men and women (peptic ulcer disease: *P* = .001; remaining 20 diseases *P* < .001), with the exception of anemia (*P* = .27). The prevalences of all chronic diseases were significantly different among the age groups for each sex (*P* < .001). Value is percentage.

**Table:** 

Rank	Three-Way Patterns Of Chronic Disease	n (%)	Age, y	%	Number of Chronic Diseases, Mean (SD)	Number of Outpatient Facilities Visited, Mean (SD)	Number of Admissions, Mean (SD)	Total Outpatient Medical Expenditure (US$), Mean (SD)
1	Hypertension, peptic ulcer disease, coronary heart disease	62,480 (12.4)	75–79	11.0	6.5 (1.7)	3.6 (2.1)	0.7 (1.2)	7,208 (8,345)
			80–84	13.8				
			85–89	14.2				
			90–94	13.0				
			95–99	10.4				
			≥100	7.4				
2	Hypertension, dyslipidemia, peptic ulcer disease	55,741 (11.0)	75–79	11.1	6.4 (1.7)	3.6 (2.1)	0.6 (1.1)	6,464 (6,726)
			80–84	12.1				
			85–89	10.4				
			90–94	7.5				
			95–99	4.3				
			≥100	2.9				
3	Hypertension, dyslipidemia, coronary heart disease	54,460 (10.8)	75–79	10.6	6.3 (1.8)	3.5 (2.0)	0.6 (1.1)	6,211 (6,645)
			80–84	11.8				
			85–89	10.7				
			90–94	8.0				
			95–99	4.9				
			≥100	2.4				
4	Hypertension, peptic ulcer disease, cerebrovascular accident	46,798 (9.3)	75–79	7.9	6.5 (1.8)	3.7 (2.1)	0.7 (1.2)	6,783 (7,226)
			80–84	10.4				
			85–89	11.4				
			90–94	10.5				
			95–99	8.6				
			≥100	6.6				
5	Hypertension, peptic ulcer disease, urologic disease	41,346 (8.2)	75–79	6.7	6.6 (1.8)	4.1 (2.2)	0.7 (1.2)	7,100 (5,971)
			80–84	9.4				
			85–89	10.4				
			90–94	10.1				
			95–99	7.6				
			≥100	6.2				
6	Dyslipidemia, peptic ulcer disease, coronary heart disease	41,263 (8.2)	75–79	8.0	6.6 (1.7)	3.7 (2.1)	0.7 (1.2)	6,861 (7,345)
			80–84	9.1				
			85–89	8.1				
			90–94	6.1				
			95–99	3.6				
			≥100	2.0				
7	Hypertension, peptic ulcer disease, cancer	38,295 (7.6)	75–79	6.7	6.5 (1.8)	3.9 (2.1)	1.0 (1.4)	8,035 (8,193)
			80–84	8.8				
			85–89	8.5				
			90–94	7.2				
			95–99	5.1				
			≥100	3.4				
8	Hypertension, dyslipidemia, cerebrovascular accident	37,825 (7.5)	75–79	7.0	6.4 (1.8)	3.6 (2.1)	0.6 (1.1)	6,052 (5,851)
			80–84	8.5				
			85–89	8.0				
			90–94	5.9				
			95–99	3.7				
			≥100	2.2				
9	Hypertension, cerebrovascular accident, coronary heart disease	37,755 (7.5)	75–79	6.0	6.7 (1.8)	3.7 (2.1)	0.7 (1.2)	6,877 (7,586)
			80–84	8.5				
			85–89	9.7				
			90–94	9.4				
			95–99	8.2				
			≥100	5.4				
10	Hypertension, peptic ulcer disease, osteoarthritis/spine disorder	37,352 (7.4)	75–79	6.6	6.6 (1.8)	4.4 (2.3)	0.7 (1.1)	7,400 (7,316)
			80–84	8.4				
			85–89	8.3				
			90–94	7.3				
			95–99	5.5				
			≥100	4.0				
11	Hypertension, peptic ulcer disease, hyperuricemia	33,579 (6.6)	75–79	6.3	6.6 (1.8)	3.5 (2.0)	0.7 (1.2)	6,694 (7,121)
			80–84	7.3				
			85–89	6.8				
			90–94	6.1				
			95–99	4.5				
			≥100	2.5				
12	Hypertension, urologic disease, coronary heart disease	33,331 (6.6)	75–79	5.1	6.8 (1.8)	4.0 (2.2)	0.7 (1.2)	7,006 (6,557)
			80–84	7.6				
			85–89	9.1				
			90–94	8.7				
			95–99	7.1				
			≥100	4.9				
13	Hypertension, dyslipidemia, diabetes mellitus	33,067 (6.5)	75–79	7.4	6.2 (1.9)	3.2 (1.9)	0.5 (1.0)	6,373 (6,328)
			80–84	6.7				
			85–89	4.8				
			90–94	2.6				
			95–99	1.5				
			≥100	0.5				
14	Hypertension, dyslipidemia, urologic disease	31,911 (6.3)	75–79	5.7	6.6 (1.9)	3.9 (2.2)	0.5 (1.0)	6,519 (5,078)
			80–84	7.3				
			85–89	7.0				
			90–94	5.4				
			95–99	3.4				
			≥100	1.9				
15	Hypertension, dyslipidemia, hyperuricemia	31,777 (6.3)	75–79	6.6	6.2 (1.9)	3.3 (2.0)	0.5 (1.0)	5,696 (5,722)
			80–84	6.7				
			85–89	5.3				
			90–94	4.0				
			95–99	2.0				
			≥100	1.5				

**Table:** 

Rank	3-way patterns of chronic disease	n (%)	Age, y	%	Number of chronic diseases Mean (SD)	Number of outpatient facilities visited Mean (SD)	Number of admissions Mean (SD)	Total outpatient medical expenditure (US$) Mean (SD)
1	Hypertension, Dyslipidemia, Peptic ulcer disease	102,907 (12.8)	75–79	12.5	6.0 (1.7)	3.8 (2.1)	0.4 (0.9)	5,871 (4,944)
			80–84	14.9				
			85–89	13.1				
			90–94	8.9				
			95–99	5.2				
			≥100	2.6				
2	Hypertension, Peptic ulcer disease, Osteoarthritis/Spine disorder	90,394 (11.2)	75–79	10.0	6.2 (1.7)	4.3 (2.2)	0.5 (1.0)	6,551 (5,342)
			80–84	13.3				
			85–89	12.3				
			90–94	9.5				
			95–99	6.8				
			≥100	3.8				
3	Hypertension, Dyslipidemia, Osteoarthritis/Spine disorder	85,869 (10.7)	75–79	10.9	6.0 (1.8)	4.2 (2.1)	0.4 (0.8)	5,848 (4,148)
			80–84	12.6				
			85–89	10.2				
			90–94	6.1				
			95–99	3.4				
			≥100	1.2				
4	Hypertension, Peptic ulcer disease, Coronary heart disease	81,226 (10.1)	75–79	7.8	6.3 (1.7)	3.8 (2.2)	0.6 (1.1)	6,462 (6,530)
			80–84	11.3				
			85–89	12.7				
			90–94	12.1				
			95–99	9.9				
			≥100	6.7				
5	Hypertension, Dyslipidemia, Coronary heart disease	78,426 (9.7)	75–79	8.9	6.1 (1.8)	3.7 (2.1)	0.4 (0.9)	5,627 (5,068)
			80–84	11.2				
			85–89	11.0				
			90–94	8.0				
			95–99	5.1				
			≥100	2.4				
6	Hypertension, Insomnia, Peptic ulcer disease	64,299 (8.0)	75–79	6.9	6.4 (1.7)	4.1 (2.3)	0.6 (1.0)	6,629 (6,021)
			80–84	9.3				
			85–89	9.0				
			90–94	7.6				
			95–99	5.7				
			≥100	3.5				
7	Hypertension, Peptic ulcer disease, Cerebrovascular accident	63,959 (7.9)	75–79	5.9	6.4 (1.8)	3.9 (2.2)	0.6 (1.0)	6,228 (5,518)
			80–84	9.0				
			85–89	10.3				
			90–94	9.4				
			95–99	7.9				
			≥100	5.4				
8	Dyslipidemia, Peptic ulcer disease, Osteoarthritis/Spine disorder	61,579 (7.6)	75–79	8.0	6.5 (1.7)	4.5 (2.3)	0.4 (0.9)	6,455 (4,639)
			80–84	9.1				
			85–89	7.0				
			90–94	4.1				
			95–99	2.2				
			≥100	0.9				
9	Hypertension, Osteoporosis, Osteoarthritis/Spine disorder	60,609 (7.5)	75–79	6.5	6.1 (1.8)	4.4 (2.2)	0.4 (0.8)	6,236 (4,387)
			80–84	9.3				
			85–89	8.6				
			90–94	5.9				
			95–99	3.3				
			≥100	1.3				
10	Hypertension, Insomnia, Dyslipidemia	60,545 (7.5)	75–79	7.5	6.2 (1.8)	4.0 (2.2)	0.4 (0.9)	5,729 (4,769)
			80–84	8.9				
			85–89	7.4				
			90–94	4.7				
			95–99	2.6				
			≥100	1.3				
11	Hypertension, Dyslipidemia, Cerebrovascular accident	60,292 (7.5)	75–79	6.6	6.2 (1.8)	3.8 (2.2)	0.4 (0.9)	5,572 (4,436)
			80–84	8.8				
			85–89	8.6				
			90–94	6.1				
			95–99	4.1				
			≥100	1.8				
12	Hypertension, Osteoporosis, Peptic ulcer disease	58,183 (7.2)	75–79	6.0	6.4 (1.7)	4.2 (2.2)	0.5 (0.9)	6,509 (4,829)
			80–84	8.8				
			85–89	8.6				
			90–94	6.6				
			95–99	3.7				
			≥100	1.6				
13	Hypertension, Osteoarthritis/Spine disorder, Coronary heart disease	57,035 (7.1)	75–79	5.7	6.5 (1.7)	4.3 (2.2)	0.5 (1.0)	6,542 (5,749)
			80–84	8.3				
			85–89	8.6				
			90–94	7.1				
			95–99	5.6				
			≥100	3.1				
14	Hypertension, Osteoporosis, Dyslipidemia	56,245 (7.0)	75–79	6.5	6.1 (1.8)	4.0 (2.1)	0.3 (0.8)	5,863 (3,904)
			80–84	8.6				
			85–89	7.5				
			90–94	4.6				
			95–99	2.1				
			≥100	0.9				
15	Dyslipidemia, Peptic ulcer disease, Coronary heart disease	56,085 (7.0)	75–79	6.4	6.5 (1.7)	3.9 (2.2)	0.5 (1.0)	6,296 (5,739)
			80–84	8.1				
			85–89	7.7				
			90–94	5.6				
			95–99	3.1				
			≥100	1.7				

Multinomial logistic regression analysis indicated that the co-occurrence of 3 or more diseases increased with increasing age until approximately age 90, and decreased thereafter (Table 3). Women were less likely to have 3 or more co-occurring diseases. Home medical care service users were more likely to have 3 or more co-occurring diseases than nonusers were. The co-occurrence of 3 or more diseases was positively associated with the number of outpatient facilities visited and hospital admissions.

**Table 3:** Association of Sociodemographic and Medical Variables With the Prevalence of 3 or More Co-Occurring Chronic Diseases Among Participants (N = 1,311,116), Study of Patterns of Co-Occurrence of Chronic Diseases Among People Aged ≥75 in Tokyo, Japan, September 2013–August 2014

Characteristic	Number of Diseases						
	None, % (n = 99,981)	≤2, % (n = 363,831	≥3, % (n = 847,304)	≤2 Versus None^a^		≥3 Versus None^a^	
				OR (95% CI)	aOR (95% CI)^b^	OR (95% CI)	aOR (95% CI)^b^
**Sex**							
Men	37.3	37.7	39.0	1 [Reference]	1 [Reference]	1 [Reference]	1 [Reference]
Women	62.7	62.3	61.0	0.98 (0.97–1.00)	0.92 (0.91–0.94)	0.93 (0.92–0.94)	0.83 (0.82–0.84)
**Age, y**							
75–79	52.2	50.9	42.5	1 [Reference]	1 [Reference]	1 [Reference]	1 [Reference]
80–84	23.5	25.7	30.8	1.12 (1.10–1.14)^a^	1.10 (1.08–1.12)^a^	1.61 (1.59–1.64)^a^	1.53 (1.51–1.56)^a^
85–89	13.6	14.2	18.0	1.07 (1.05–1.09)^a^	1.09 (1.07–1.11)^a^	1.62 (1.59–1.66)^a^	1.65 (1.62–1.69)^a^
90–94	7.2	6.5	6.8	0.93 (0.91–0.96)^a^	0.99 (0.96–1.02)	1.16 (1.13–1.19)^a^	1.30 (1.27–1.34)^a^
95–99	2.8	2.2	1.6	0.80 (0.76–0.83)^a^	0.86 (0.82–0.90)^a^	0.69 (0.66–0.72)^a^	0.80 (0.76–0.84)^a^
≥100	0.7	0.5	0.2	0.67 (0.61–0.73)^a^	0.73 (0.67–0.80)^a^	0.36 (0.33–0.39)^a^	0.45 (0.41–0.49)^a^
**Household income**							
Nonhigh household income	85.4	85.6	85.7	1 [Reference]	1 [Reference]	1 [Reference]	1 [Reference]
High household income	14.6	14.4	14.3	0.99 (0.97–1.01)	0.91 (0.90–0.93)^a^	0.98 (0.96–1.00)	0.84 (0.82–0.86)^a^
Use of home medical care services							
No	96.7	93.9	91.2	1 [Reference]	1 [Reference]	1 [Reference]	1 [Reference]
Yes	3.3	6.1	8.8	1.87 (1.81–1.95)^a^	2.13 (2.05–2.22)^a^	2.81 (2.72–2.91)^a^	3.00 (2.89–3.11)^a^
**Number of outpatient facilities visited**							
1	50.1	27.9	13.5	1.00	1.00	1.00	1.00
2	26.9	29.1	21.7	1.94 (1.91–1.98)^a^	1.95 (1.92–1.98)^a^	3.00 (2.95–3.05)^a^	3.00 (2.95–3.05)^a^
3	13.4	20.5	21.8	2.74 (2.68–2.79)^a^	2.76 (2.71–2.82)^a^	6.03 (5.91–6.15)^a^	6.08 (5.95–6.20)^a^
4	5.9	11.8	17.1	3.61 (3.50–3.71)^a^	3.66 (3.56–3.77)^a^	10.77 (10.47–11.07)^a^	10.90 (10.59–11.21)^a^
≥5	3.7	10.7	25.8	5.23 (5.05–5.42)^a^	5.36 (5.17–5.56)^a^	26.15 (25.27–27.06)^a^	26.69 (25.59–27.53)^a^
**Number of hospital admissions**							
0	86.1	84.5	73.1	1 [Reference]	1 [Reference]	1 [Reference]	1 [Reference]
1	9.6	10.4	16.4	1.11 (1.08–1.14)^a^	0.97 (0.95–0.99)^a^	2.01 (1.97–2.06)^a^	1.50 (1.47–1.54)^a^
2	2.9	3.2	6.1	1.13 (1.08–1.17)^a^	0.99 (0.95–1.04)	2.47 (2.38–2.57)^a^	1.86 (1.79–1.93)^a^
≥3	1.5	1.8	4.4	1.27 (1.20–1.35)^a^	1.13 (1.06–1.19)^a^	3.60 (3.41–3.79)^a^	2.72 (2.58–2.87)^a^

Abbreviations: aOR, adjusted odds ratio; CI, confidence interval; OR, odds ratio.

a Significant (*P* < .05).

b Adjusted for all other variables.

## Discussion

By using a large-scale medical claims database, we examined the common 3-way chronic disease patterns among all Tokyo residents aged 75 or older who received medical care. Approximately two-thirds of residents had recorded diagnoses of 3 or more co-occurring chronic diseases during the study period. Common 3-way disease patterns varied by sex and age group, where the prevalence of 3 or more co-occurring chronic diseases was higher in men and in people aged 85 to 89, but lower in people aged 90 or older. The number of outpatient facilities visited and hospital admissions was positively associated with the co-occurrence of 3 or more chronic diseases.

The high prevalence of 3 or more co-occurring diseases in our study sample is similar to findings from previous studies conducted in the United States and Europe (3,11–13). However, Steinman et al reported that approximately 90% of veterans aged 75 or older had 3 or more co-occurring diseases, a difference which may be explained by the greater burden of illness among US veterans than among nonveterans of a similar age (8). That study also detected variations in common 3-way disease patterns according to sex and age (8). The development of multimorbidity management guidelines that account for these variations may help to determine safer and more effective approaches for specific disease patterns (8), though the concurrent treatment of multiple diseases is accompanied by an increased risk of adverse drug events. Clinician judgement is therefore needed to make appropriate treatment decisions that minimize this risk; however, current clinical guidelines are unable to sufficiently inform this process. Furthermore, it is difficult to integrate various paper-based disease-specific guidelines to comprehensively document all possible adverse drug events (7). Clinical guidelines and treatment planning tools that alert physicians to possible adverse drug events associated with multimorbidity patterns may assist clinical judgement (7). It may also be useful to develop electronic medical records that integrate treatment recommendations for patients with multiple disorders.

The prevalence of 3 or more co-occurring chronic diseases was higher in men in our study, which differed from the results of studies on representative samples in the United States and Europe (3,18). The association between multimorbidity and sex may be dependent on the methods for disease identification, such as eligibility conditions and coding systems (4,5,8,11). For example, our inclusion of conditions that are more common in men (such as hyperuricemia and urologic disease), which were not examined in previous studies (3,18), may have contributed to these differences.

Our study found that the prevalence of 3 or more co-occurring chronic diseases increased with increasing age until approximately age 90, but decreased thereafter. Previous studies did not examine the association between aging and the prevalence of multimorbidity in people aged 90 or older because they had collectively analyzed people aged 85 or older as a single age group (3,5,8). We sought to elucidate this association by further dividing these patients into 3 age groups. We used these age groups because approximately 10% of patients aged 75 or older in this study were actually aged 90 or older, and the average life expectancy in Japan has become closer to 90 (19). Moreover, this negative association of older age with multimorbidity may be partially explained by the survival effect and the disease identification method used in this study. The prevalence of 3 or more co-occurring chronic diseases may have decreased with increasing age in these older patients because a large proportion of older adults with multimorbidity died at an earlier age (6). Furthermore, the prevalence of the target chronic diseases may have been underestimated among adults aged 90 or older; those adults are less likely to receive drugs to treat chronic diseases because of their lower need-for-treatment criteria (20). Therefore, our results may reflect a shift in emphasis from the curing of chronic diseases to their management with consideration of the quality of life in these older adults.

We observed variations in total outpatient medical expenditure among different patterns of chronic diseases. Among the common 3-way disease patterns, the combination of hypertension, peptic ulcer disease, and cancer had the highest expenditure in men ($8,035; 7th in order of prevalence) and women ($7,330; 38th in order of prevalence). Cancer is one of the costliest diseases for patients (21), and its inclusion in disease patterns with high costs is therefore unsurprising. Further studies are needed to identify the multimorbidity patterns that require high expenditures and to better understand the growing population of high-cost and high-need individuals.

People with multimorbidity were more likely to have high numbers of visits to outpatient facilities and hospital admissions as previously reported (5). The management of these patients can be complicated because the risk of adverse drug events is higher when patients receive medical care from different physicians and institutions. The number of outpatient facilities visited increased with an increasing prevalence of 3 or more co-occurring chronic diseases and varied among different patterns of multimorbidity: the number was highest for people of either sex with co-occurring hypertension, peptic ulcer disease, and osteoarthritis/spine disorder. The number of outpatient visits in Japan has been reported to be higher than that of other Organisation for Economic Co-operation and Development countries, which may be due to the free access characteristic of Japan’s health care system (22,23). Under this system, people are able to seek care at any institution, which has resulted in widespread access to treatments that prevent chronic diseases (22). However, this has also encouraged “doctor shopping” and duplicate patient visits (23,24), which elevate the risk of duplicative prescriptions (16,25) and adverse drug events (24).

The increased use of home medical care services by patients with multiple disorders may indicate a higher risk of functional decline, because users of these services are generally unable to travel to medical facilities for nonemergency treatment. A previous study reported multimorbidity to be associated with functional decline (26), which is also associated with an elevated risk of hospital admissions and readmissions in adults aged 65 or older (27,28). A strategy is therefore needed to prevent worsening conditions in these patients.

A strength of this study is the use of health insurance claims data from over 1.3 million Japanese people aged 75 or older. The results reported here are representative of citizens aged 75 or older living in Tokyo, because we include data from 97.1% of Tokyo citizens in that age group (1,311,116 of the 1,350,964 people insured by the program in September 2014). Our study included people with disabilities, those living in nursing homes, and oldest-old adults (≥90 y), who are frequently excluded from field studies (13,17). Thus, the results obtained from this study can be generalized to other adults aged 75 or older living in urban areas in Japan. Another strength was the quantification of the prevalence of common 3-way patterns according to sex and age group.

This study had several limitations. First, the prevalence of peptic ulcer disease may have been overestimated in our analysis, because physicians in Japan often record diagnoses of peptic ulcer disease to justify the administration of drugs to alleviate gastrointestinal symptoms resulting from the use of nonsteroidal anti-inflammatory drugs (29). Second, despite efforts to minimize errors in disease identification by using ICD-10 codes and prescribed drugs, the diagnoses in the claims data were not clinically verified by specialists (13,17). Moreover, cancer, cerebrovascular accidents, and coronary heart disease were identified by using only ICD-10 codes. These 3 diseases had a higher prevalence in our study than was reported in a Japanese national survey (30), although that survey identified diseases by using only the recorded main diagnoses and may therefore have underestimated prevalence. Third, we were unable to consider variations in disease burden because of the lack of disease severity information in Japanese claims data. Because disease severity can directly affect treatment approaches and expenditures, this variable should be considered in future analyses of multimorbidity patterns.

Identifying the common 3-way disease patterns in older patients may support the development of clinical guidelines for multimorbidity. Moreover, the high prevalence of multimorbidity and its association with the use of multiple medical institutions indicate that patients with multiple disorders are more vulnerable to duplicative prescriptions and adverse drug events. It is therefore important to implement systemic changes to consolidate and share patient information on prescriptions and visits to other medical facilities.

## Post-Test Information

To obtain credit, you should first read the journal article. After reading the article, you should be able to answer the following, related, multiple-choice questions. To complete the questions (with a minimum 75% passing score) and earn continuing medical education (CME) credit, please go to http://www.medscape.org/journal/pcd. Credit cannot be obtained for tests completed on paper, although you may use the worksheet below to keep a record of your answers.

You must be a registered user on http://www.medscape.org. If you are not registered on http://www.medscape.org, please click on the “Register” link on the right hand side of the website.

Only one answer is correct for each question. Once you successfully answer all post-test questions, you will be able to view and/or print your certificate. For questions regarding this activity, contact the accredited provider, CME@medscape.net. For technical assistance, contact CME@medscape.net. American Medical Association’s Physician’s Recognition Award (AMA PRA) credits are accepted in the US as evidence of participation in CME activities. For further information on this award, please go to https://www.ama-assn.org. The AMA has determined that physicians not licensed in the US who participate in this CME activity are eligible for *AMA PRA Category 1 Credits™*. Through agreements that the AMA has made with agencies in some countries, AMA PRA credit may be acceptable as evidence of participation in CME activities. If you are not licensed in the US, please complete the questions online, print the AMA PRA CME credit certificate, and present it to your national medical association for review.

## Post-Test Questions

### Study Title: Patterns of Co-Occurrence of Chronic Disease Among Older Adults in Tokyo, Japan

#### CME Questions

1. You are seeing a married woman and man, both of whom are 81 years of age, for the management of multiple chronic diseases. What was the approximate average number of chronic illnesses among women and men in the current study by Mitsutake and colleagues?
  - 2
  - 4
  - 6
  - 10
2. What was the most common combination of 3 different chronic illnesses among women in the current study?
  - Hypertension, osteoporosis, dyslipidemia
  - Hypertension, peptic ulcer disease, dyslipidemia
  - Cerebrovascular accident, coronary heart disease, peptic ulcer disease
  - Osteoarthritis/spine disorder, osteoporosis, diabetes mellitus
3. What was the most common combination of 3 different chronic illnesses among men in the current study?
  - Hypertension, dyslipidemia, diabetes mellitus
  - Hypertension, cerebrovascular accident, coronary heart disease
  - Urologic disease, dyslipidemia, cancer
  - Hypertension, peptic ulcer disease, coronary heart disease
4. The couple wants to know about why they have multiple chronic illnesses. What should you consider regarding trends in chronic illness in the current study as you speak with them?
  - There was a linear association of more chronic illness as age increased
  - The rate of having 3 or more chronic diseases decreased after age 90 years
  - Women were more likely than men to have at least 3 chronic illnesses
  - Having multiple chronic illnesses was not associated with increased use of health care resources
